# Novel clinical features of nonconvulsive status epilepticus

**DOI:** 10.12688/f1000research.10939.1

**Published:** 2017-09-15

**Authors:** Masao Nagayama, Sunghoon Yang, Romergryko G. Geocadin, Peter W. Kaplan, Eisei Hoshiyama, Azusa Shiromaru-Sugimoto, Mitsuru Kawamura

**Affiliations:** 1 Department of Neurology, International University of Health and Welfare School of Medicine, Narita, Chiba, Japan; 2 Department of Neurology and the Center for Stroke and Neurocritical Care, International University of Health and Welfare Atami Hospital, Atami, Shizuoka, Japan; 3 Departments of Neurology, Anesthesiology and Critical Care, Neurosurgery, and Medicine, Division of Neurosciences Critical Care, The Johns Hopkins University School of Medicine, Baltimore, Maryland, USA; 4 Department of Neurology, Johns Hopkins University School of Medicine, Baltimore, Maryland, USA; 5 Department of Emergency and Critical Care Medicine and Neurology, Dokkyo Medical University, Mibu, Tochigi, Japan; 6 Department of Neurology, Showa University School of Medicine, Shinagawa-ku, Tokyo, Japan

**Keywords:** epilepsy, nonconvulsive status epilepticus, electroencephalography

## Abstract

Nonconvulsive status epilepticus (NCSE) has rapidly expanded from classical features such as staring, repetitive blinking, chewing, swallowing, and automatism to include coma, prolonged apnea, cardiac arrest, dementia, and higher brain dysfunction, which were demonstrated mainly after the 2000s by us and other groups. This review details novel clinical features of NCSE as a manifestation of epilepsy, but one that is underdiagnosed, with the best available evidence. Also, we describe the new concept of epilepsy-related organ dysfunction (Epi-ROD) and a novel electrode and headset which enables prompt electroencephalography.

## Introduction

Most clinicians and laypeople equate an epileptic attack with convulsive seizures. Nonconvulsive seizures and nonconvulsive status epilepticus (NCSE)—a serious condition of prolonged or recurrent nonconvulsive epileptic attacks—are often not recognized, even by specialists in the fields of intensive care, neurology, neurosurgery, and epileptology ^1, 2^. In daily clinical practice, nonconvulsive seizures and NCSE are rarely included in differential diagnoses, even by those who recognize the underlying concepts. Even if they are included, practitioners often fail to go beyond identifying complex partial seizures or absence seizures.

This review describes NCSE especially as a manifestation of epileptic seizure that has been mainly elucidated since about 2000 but is often underdiagnosed despite its treatable nature.

## Nonconvulsive status epilepticus and its causes

Epileptic seizures include overt seizures such as generalized convulsive seizures, those without convulsions involving impairment of consciousness such as complex partial seizures, and those that are usually perceptible only to the patient such as sensory or psychological seizures (simple, partial seizures). NCSE is thought to arise from simple or complex partial seizures or from generalized atypical or atypical absence seizures that persist or recur for at least 30 minutes. However, in 2012, the Neurocritical Care Society defined status epilepticus (SE) as five minutes or more of continuous clinical and/or electrographic seizure or recurrent seizure activity without recovery between seizures ^3^. In 2015, the International League Against Epilepsy (ILAE) issued a new SE classification, including a detailed semiologic axis ^4^. In this classification, NCSE was classified into “NCSE with coma” and “NCSE without coma”. “NCSE without coma” was subclassified into “generalized”, “focal”, and “unknown whether focal or generalized”. Also, it should be noted that focal lesions in focal or secondarily generalized NCSE involve not only the temporal lobes but also the frontal, parietal, and occipital lobes. A separate manifestation of “NCSE in coma” has been increasingly identified since the advent of continuous EEG monitoring after cardio-respiratory arrest (CRA).

**Figure 1.**
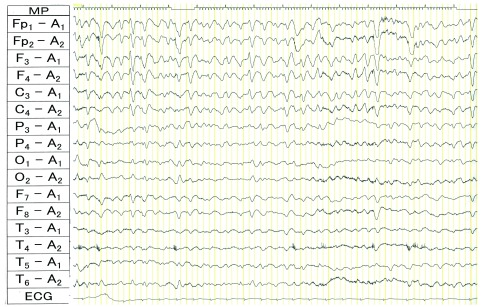
Ictal electroencephalogram (EEG) in a patient with nonconvulsive status epilepticus (NCSE). Ictal EEG of patient #8 in Table 1. This EEG was taken from an 83-year-old female with acute hyperammonemic encephalopathy resulting from Osler–Weber–Rendu disease. During the episode of NCSE with total aphasia, triphasic wave-like waves were observed with spatiotemporal evolution. Five minutes after starting phenytoin, aphasia started to improve, which completely disappeared 15 minutes after starting phenytoin.

NCSE has diverse causes such as acute encephalopathy, cerebrovascular diseases (18–29% of hemorrhagic cases were reported to have caused NCSE), central nervous system (CNS) infection, brain tumor, traumatic brain injury, and postoperative complications ^5^. In 40 NCSE patients, determined by a combination of the EEG waveform changes and the corresponding clinical signs and symptoms and treated in our department (out of 1,116 serial cases from May 2006 to September 2014), major underlying conditions were, in order of frequency, acute encephalopathy (eight cases), cerebrovascular disease (eight cases), cardiac disease (six cases), CNS infection (five cases), chronic alcohol dependence (four cases), degenerative neurological diseases, traumatic CNS injury, no underlying condition, malignant disease, atrial fibrillation, and renal disease (three cases, respectively), and epilepsy (two cases) (see Table 1 and Figure 1). Given that acute encephalopathy, the most frequent cause, is often accompanied by diverse and serious neurological symptoms such as impaired consciousness level, mental alteration, and SE (convulsive and nonconvulsive), it is necessary to take a careful history and physical findings to comprehend and differentiate the underlying pathology. We need to be aware that epileptic seizure can coexist with acute encephalopathy and that NCSE and convulsive seizure can coexist in a patient. For details on the causes of NCSE, please refer to the monograph edited by Kaplan and Drislane ^6^.

**Table 1.** Clinical features of patients with nonconvulsive status epilepticus. 5-FU, 5-fluorouracil; CP, complex partial; DIC, disseminated intravascular coagulation; GCSE, generalized convulsive status epilepticus; ICH, intracerebral hemorrhage; HSV, herpes simplex virus; NCSE, nonconvulsive status epilepticus; SAH, subarachnoid hemorrhage.

#	Age	Sex	Major underlying disease(s)	Major clinical features	Convulsion	Type of status epilepticus	Organ dysfunction before onset	Organ dysfunction complicated	Outcome
1	53	F	Acute water intoxication, chronic alcohol dependence	Acute encephalopathy, alteration of consciousness	Yes	NCSE/CP + GCSE	Rhabdomyolysis	None	Recovery
2	89	M	Bilateral intracranial internal carotid artery stenosis	Mimetic facial automatism	None	NCSE/CP	None	None	Recovery
3	65	M	Frontotemporal lobar degeneration	Klüver–Bucy syndrome	None	NCSE/CP	None	None	Recovery
4	79	M	Cerebrovascular dementia	Loss of consciousness attacks	None	NCSE/absence?	Chronic renal failure, old myocardial infarction	None	Recovery
5	76	M	Acute 5-FU encephalopathy	Coma	None	NCSE/CP	Liver dysfunction	None	Recovery
6	35	F	Acute HSV encephalitis	Prolonged post- hyperventilation apnea, overeating	Yes	NCSE/CP	None	Acute respiratory failure	Refractory
7	56	M	Acute hepatic encephalopathy, alcoholic	Alteration of consciousness	Yes	NCSE/CP	None	Takotsubo cardiomyopathy	Recovery
8	83	F	Acute hyperammonemic encephalopathy, Osler’s disease	Total aphasia	None	NCSE/CP	Valvular heart disease, atrial fibrillation	Renal dysfunction	Recovery
9	82	M	Old cerebral infarction, extracranial internal carotid artery stenosis	Broca’s aphasia	None	NCSE/CP	Chronic pancreatitis	QT prolongation	Recovery
10	83	M	None	Alteration of consciousness	Yes	NCSE/CP + GCSE	None	Renal dysfunction	Refractory
11	76	F	Acute artery-to-artery cerebral embolism	Loss of consciousness attacks, alteration of consciousness	Yes	NCSE/CP	None	Cardiopulmonary arrest	Death
12	69	F	Atrial fibrillation	Staring, amnesia, alteration of consciousness	Yes	NCSE/CP	None	None	Recovery
13	79	F	Familial Parkinson’s disease	Loss of consciousness attacks	None	NCSE/CP	None	None	Sudden death
14	77	F	Traumatic brain injury	Dementia, depression, staring, automatism, tremor	None	NCSE/CP	None	None	Recovery
15	57	M	Acute hepatic encephalopathy, alcoholic	Automatism at right arm	None	NCSE/CP	Hypothyroidism	None	Recovery
16	60	M	SAH and postoperative meningoencephalitis	Central alveolar hypoventilation	None	NCSE/CP	None	Central alveolar hypoventilation, pneumonia	Recovery
17	74	M	Cerebral sinus occlusion and reversible posterior leukoencephalopathy syndrome	Hallucination, abnormal behavior	None	NCSE/CP	None	None	Recovery
18	20	M	Traumatic cervical injury	Recent memory disturbance, word-finding difficulty	None	NCSE	Hyperthyroidism	None	Recovery
19	73	M	Infective endocarditis	Consciousness disturbance	None	NCSE	None	None	Recovery
20	55	M	Acute encephalopathy, chronic renal failure	Alteration of consciousness	None	NCSE	Chronic renal failure	None	Recovery
21	77	M	Acute encephalopathy, sepsis	Alteration of consciousness, Broca’s aphasia	None	NCSE	Chronic hepatitis	None	Recovery
22	56	M	Acute encephalopathy	Higher brain dysfunction	None	NCSE	Chronic renal failure	None	Recovery
23	79	M	Chronic heart failure, atrial fibrillation	Consciousness disturbance	None	NCSE	None	None	Recovery
24	89	M	Chronic kidney disease, chronic heart failure	Alteration of consciousness, automatism	None	NCSE	Chronic kidney disease, heart failure	Chronic kidney disease	Refractory
25	77	M	Chronic renal failure, postoperative bladder carcinoma	Alteration of consciousness	None	NCSE	Chronic renal failure	DIC	Death
26	76	M	Chronic kidney disease, atrial fibrillation	Loss of consciousness attacks	None	NCSE	Renal dysfunction	Severe bradycardia	Recovery
27	89	F	Femoral head fracture	Alteration of consciousness	None	NCSE	None		Recovery
28	68	F	Post-traumatic epilepsy	Alteration of consciousness, auditory hallucination, delusion of persecution	Yes	NCSE/CP + GCSE	Giant liver hemangioma	None	Recovery (recurred)
29	65	M	Alcohol dependence	Alteration of consciousness	Yes	NCSE/CP + GCSE	None	Acute prerenal renal failure	Recovery
30	18	F	Non-HSV encephalitis sequelae	Prolonged post- hyperventilation apnea	Yes	NCSE/CP + GCSE	None	Multiple organ failure	Death
31	56	M	Intravascular malignant lymphomatosis	Alteration of consciousness	Yes	NCSE/CP + GCSE	None	None	Recovery
32	51	M	Postoperative SAH	Alteration of consciousness	Yes	NCSE/CP + GCSE	None	None	Recurrent
33	49	M	Acute disseminated encephalomyelitis	Alteration of consciousness	Yes	NCSE/CP + GCSE	None	Renal and liver dysfunction	Recurrent
34	15	M	None	Consciousness disturbance, four extremities weakness	Yes	NCSE/CP + GCSE	None	None	Recovery
35	45	F	Epilepsy, central nervous system lupus, lupus nephritis	Dysgraphia	Yes	NCSE/CP + GCSE	None	None	Recovery
36	86	M	None	Difficulties in speaking, facial twitching	None	NCSE/CP + GCSE	None	None	Recovery
37	47	M	Acute hypoglycemic encephalopathy	Consciousness disturbance	Yes	NCSE/CP + GCSE	Liver dysfunction	Renal dysfunction	Recovery
38	34	M	Acute lymphocytic leukemia, post-irradiation basal ganglia calcification	Alteration of consciousness, irritability, perseveration	Yes	NCSE/CP + GCSE	Hypothyroidism	None	Refractory
39	64	M	Meningoencephalitis	Alteration of consciousness	Yes	NCSE/CP + GCSE	None	Multiple organ failure, DIC, central diabetes insipidus	Death
40	78	M	Sepsis, cerebral infarction	Consciousness disturbance	Yes	NCSE/CP + GCSE	Chronic myeloid leukemia, liver dysfunction	DIC, renal dysfunction	Death

M: male, F: female, ICH: intracerebral hemorrhage, 5-FU: 5-fluorouracil, HSV: herpes simplex virus, SAH: subarachnoid hemorrhage, DIC: disseminated intravascular coagulation

## Widening the clinical spectrum of nonconvulsive status epilepticus

It is generally understood that consciousness disturbance manifests as two types: 1) depression in level of consciousness (decreased responsiveness) and 2) alteration in type of consciousness (the content of consciousness) (see Table 2). Since generalized convulsive seizures are usually accompanied by type one consciousness problems, clinicians may dismiss the possibility of epileptic attack in type two patients. However, while consciousness may continue in authentic simple partial seizures and in complex partial seizures, the content of such consciousness may still be “clouded”, especially in the latter. This is important for understanding NCSE.

**Table 2.** Expanded spectrum of manifestations of nonconvulsive status epilepticus (NCSE).

Classical clinical features Complex partial seizures type Staring, repetitive blinking, chewing, swallowing, or automatism Clouding of consciousness generally characterized by alteration of mental function consciousness with concurrent language disturbances Simple partial seizures type Symptoms linked to the anatomical and functional locations of the CNS foci Temporal lobe epilepsy, amygdalar and hippocampal lesions epigastric discomfort and uncinate fits such as autonomic seizures, psychic seizures, and parosmia Lateral temporal lesions auditory hallucinations and language disturbance Frontal lobe epilepsy motor seizures not only tonic seizures and seizures with fencing postures but also those with complex gesticulation Parietal lobe epilepsy somatosensory abnormalities such as numbness Occipital lobe epilepsy visual seizures
Consciousness disturbance Acute consciousness disturbance Comatose state Mental alteration Fluctuation of consciousness level Prolonged consciousness disturbance Protracted coma Fluctuation of consciousness level Recurrent loss of consciousness attack
Transient neurological attack (TNA) including isolated vertigo, dizziness, and headache
Higher brain dysfunction Wernicke’s aphasia, Broca’s aphasia, Klüver–Bucy syndrome Amnesia, indifference Confabulation, hallucinatory delusion, delirium Body schema disturbances (e.g. abnormal proprioception and supernumerary phantom limbs) Neglect, auditory and visual hallucinations, cortical blindness
Cognitive impairment and psychiatric manifestations Dementia, including acutedementia Abnormal behavior and/or speech Persistent laughing (status gelasticus)
Automatism Licking chops, nose wiping, facial pantomime
Abnormal eye position and movement Conjugate deviation of eyes, spontaneous nystagmus
Myoclonus of the face and extremities Especially interictal small myoclonus of the face and extremities
Autonomic dysfunction Gastrointestinal or cardiovascular autonomic events Panayiotopoulos syndrome
Acute organ dysfunction (epilepsy-related organ dysfunction [Epi-ROD]) Acute apnea, including prolonged post-hyperventilation apnea Acute cardiac arrest, acute dysfunction of other organs May cause sudden unexpected death in epilepsy (SUDEP)

In general, neurological deficits of an unexplained, episodic, fluctuating, or recurrent nature should arouse suspicion of NCSE. We need to consider convulsive SE and especially NCSE in the differential diagnosis of various acute organ dysfunctions, even in the absence of overt seizures.

### 1) Classical clinical features ^6^

It is known that complex partial seizures in cryptogenic epilepsy may manifest, for example, as staring, repetitive blinking, chewing, swallowing, or automatism but without convulsive seizures. Most cases of SE with complex partial seizures show clouding of consciousness of temporal or frontal lobe origin and are generally characterized by alteration of mental function and consciousness with concurrent language disturbances.

Simple partial SE is accompanied not by disturbance of consciousness but by clinical symptoms linked to anatomical and functional locations of CNS foci. In temporal lobe epilepsy, amygdalar and hippocampal lesions cause epigastric discomfort and uncinate fits such as autonomic seizures, psychological seizures, and parosmia, while lateral temporal lesions cause auditory hallucinations and language disturbance. Frontal lobe epilepsy manifests as motor seizures, including not only tonic seizures and seizures with fencing postures but also those with complex gesticulation. In parietal lobe epilepsy, predominant seizures are somatosensory abnormalities such as numbness, and occipital lobe epilepsy manifests as visual seizures.

### 2) Impaired level of consciousness (acute and prolonged coma)

Since about 2000, NCSE, in particular complex partial NCSE, has been identified as a cause of coma and other neurological symptoms ^6^. In a study by Towne *et al.*, at least 30 minutes of EEG monitoring identified 19 cases of NCSE (8%) out of 236 convulsion-free comatose cases admitted to the general ICU ^7^. This revealed, for the first time, the underdiagnosis of NCSE associated with coma. Accordingly, continuous EEG monitoring is now recommended, at least for patients with unexplained coma albeit without convulsions. Recognized practical criteria for EEG abnormalities in NCSE patients are thus urgently needed.

Since 2005, we have demonstrated novel treatable manifestations of NCSE including prolonged disturbance of consciousness ^8^ and prolonged post-hyperventilation apnea ^9^. Prolonged disturbance of consciousness was studied in six non-traumatic patients awakened from a coma of one month or more and with a total Glasgow coma scale score of seven or less. Two cases of NCSE were identified. One of these with symptomatic epilepsy was awakened after the start of phenytoin therapy, while the other, with viral encephalitis, was awakened after carbamazepine therapy. In case one, the estimated duration of NCSE was two weeks, and in case two it was several months ^8^.

### 3) Prolonged post-hyperventilation apnea

Healthy alert individuals with PaCO _2_ reduced by short-term hyperventilation continue to breathe regularly with a lower tidal volume until PaCO _2_ returns to normal ^10^. Post-hyperventilation apnea may also rarely occur in patients with bilateral cerebral lesions ^10, 11^. We examined a case of recurrent prolonged post-hyperventilation apnea following severe viral encephalitis in an 18-year-old female patient and identified nine previously reported cases ^9^. These 10 cases in all had the following features: year of report was 1990 or later in seven cases and onset occurred in the second decade of life in two cases, in the third decade in three cases, in the fourth decade in one case, in the fifth decade in one case, and in the sixth decade or later in three cases. Male-to-female ratio was 1:9. Associated underlying diseases were hyperventilation syndrome in five cases, severe viral encephalitis in one case, and one case each of intellectual disability, fall-induced trauma, personality/behavioral disorder, and dental caries treatment. Hyperventilation recurred in nine cases, and severe hypoxemia (SaO _2_ <80%) was observed in seven cases. The mortality rate was 30%.

**Figure 2.**
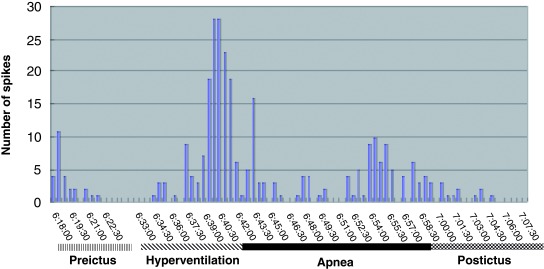
Positive spike frequency histogram. Frequency histogram analysis of positive electroencephalographic spikes in an 18-year-old woman with recurrent, prolonged, post-hyperventilation apnea. Positive spikes were marked, in particular, during hyperventilation, which was interpreted as autonomic epileptic seizures. Quoted from Nagayama ^9^.

A frequency histogram of positive EEG spikes in our own patient revealed marked positive spikes during hyperventilation episodes. These were interpreted as representing epileptic autonomic seizures ( Figure 2). Although no neurophysiological data were available for the other nine cases, given that hyperventilation attacks recurred in many of them and that involuntary movements or auras accompanied some cases, the clinical features of all cases suggested epilepsy. Therefore, we believe that prolonged post-hyperventilation apnea should properly be viewed as a novel manifestation of NCSE.

### 4) Higher brain dysfunctions and cognitive impairments

***a) Klüver–Bucy syndrome.*** Klüver–Bucy syndrome is a cluster of behavioral abnormalities resulting from temporal lobe lesions and was originally reported in a monkey model following bilateral temporal lobectomy involving the amygdalae, unci, and hippocampi. Effects include hyperoral tendencies (tendency to eat and smell everything), hypermetamorphosis (increased reaction to visual stimuli), placidity (calmness with a loss of aggression), increased sexual behavior, altered dietary preferences, hyperphagia, and pica. The syndrome seldom occurs in humans, and concurrence of all symptoms is very rare. Language and cognitive disturbances are foremost, and many cases are of a transient character. The following have been reported: bilateral temporal lesions (trauma, inflammation, cognitive disturbance, epilepsy, and cerebral infarction) and disconnection of the medial temporal lobes from cerebral, limbic, and other regions.

**Figure 3.**
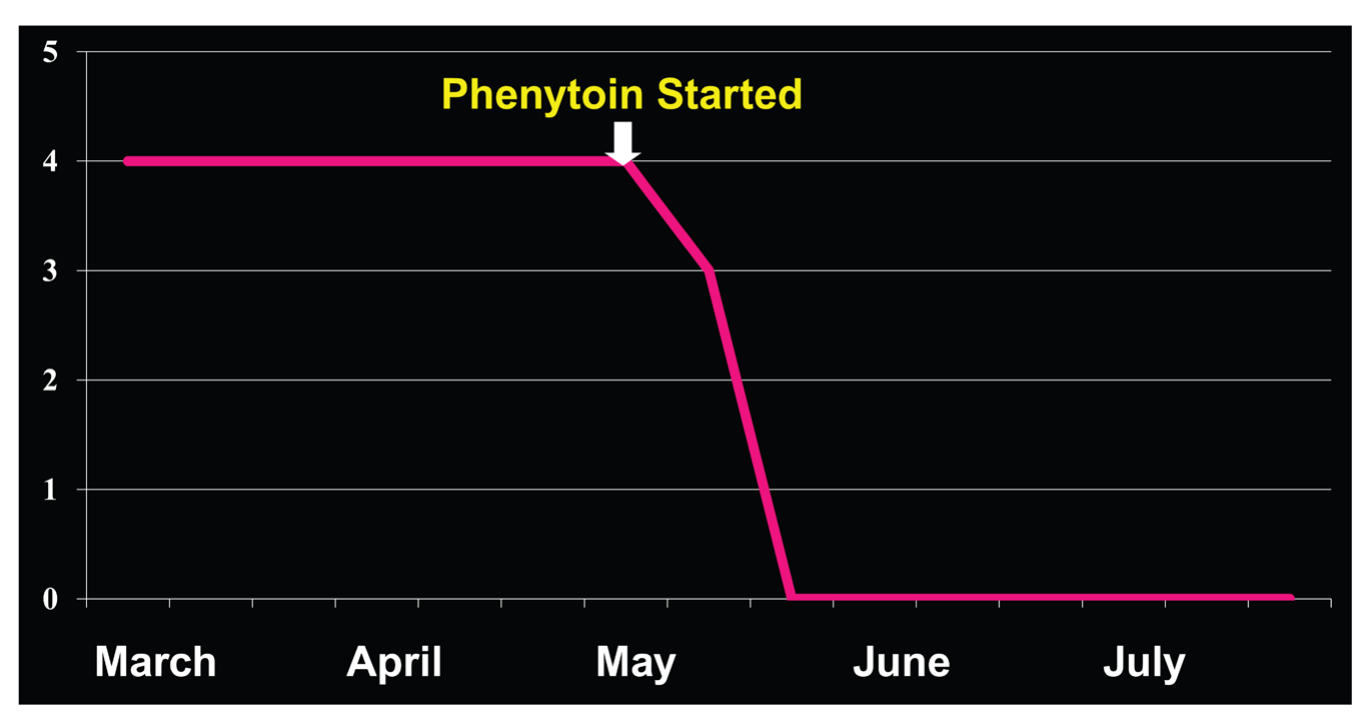
Hypersexuality in Klüver–Bucy syndrome before/after intravenous phenytoin. Changes in overeating and sexual behaviors in a 65-year-old male patient with nonconvulsive status epilepticus manifesting as Klüver–Bucy syndrome. Hypersexuality decreased immediately after the initiation of phenytoin therapy and completely disappeared two weeks later. Overeating also disappeared but was followed by severe anorexia.

With regard to our own cases, a 65-year-old male NCSE patient recovered from prolonged Klüver–Bucy syndrome in response to antiepileptic therapy ^12^. He complained chiefly of hypersexuality and gait disturbance. Aside from a history of several years of frequent nose touching resulting in skin abrasion, his presenting problems on his first visit included attacks of depressed consciousness, hypersexuality (about four episodes a day), and overeating. His past medical history was complicated and included sedative, anxiolytic, and alcohol dependence, cerebral infarction, trigeminal neuralgia, dyslalia, cognitive dysfunction, homonymous hemianopia, limb rigidity, orolingual dyskinesia, and mild bilateral incoordination. MRI revealed small, bilateral infarctions in the occipital lobes and basal ganglia and bilateral hippocampal degeneration. EEG showed repetitive synchronous grouping discharges with bilateral, fronto-parieto-temporal predominance. The findings were interpreted as NCSE manifesting as Klüver–Bucy syndrome. Phenytoin therapy was initiated (we started phenytoin because we experienced harmful effects such as glossoptosis and depressed consciousness level after benzodiazepine challenge test in other patients and because phenytoin and fosphenytoin can exert their effects rapidly, although not as much as compared with benzodiazepines), whereupon the pathological sexual behavior improved and then disappeared within two weeks ( Figure 3). Overeating also disappeared but resulted in severe anorexia. A literature survey identified two previous cases. Given the almost complete disappearance of Klüver–Bucy syndrome immediately after the initiation of phenytoin therapy and the lack of morphological abnormalities in the temporal lobes and based upon the published evidence, we consider the case as one of complex partial NCSE resulting from functional abnormalities in the temporal lobes. The case is also interesting, we believe, with regard to potential treatments for higher brain dysfunction.

***b) Other types of higher brain dysfunction.*** Epilepsy-induced higher brain dysfunctions include aphasia, amnesia, body schema disturbances (for example, abnormal proprioception and supernumerary phantom limbs), neglect, auditory and visual hallucinations, and cortical blindness.

Patients with simple, partial NCSE experience clinical symptoms corresponding to epileptogenic focal regions and present with aphasia if the focus is in the language areas. However, a premature diagnosis of aphasic seizure should be avoided because foci outside the language areas may also cause speech arrest ^13^.

As examples of higher brain dysfunctions secondary to NCSE, Midorikawa *et al.* reported cases of anterograde amnesia ^14, 15^, headache, and indifference. Cases have also been reported of Wernicke's aphasia secondary to limbic encephalitis or cerebral infarction (these remitted or disappeared in response to antiepileptic medication ^16, 17^). Other symptoms include confabulation, hallucinatory delusion, and delirium ^13, 18^. We also treated a patient with total aphasia due to NCSE secondary to hyperammonemic encephalopathy resulting from Osler–Weber–Rendu disease and a second patient with Broca's aphasia associated with NCSE secondary to right extracranial internal carotid artery stenosis ( Table 1). The first patient responded quickly to phenytoin: total aphasia remitted five minutes after phenytoin administration and completely disappeared 15 minutes later. In the second patient, Broca's aphasia disappeared spontaneously.

Although little attention has been paid to higher brain dysfunctions in connection with epilepsy, the notion of “epileptic higher brain dysfunction” needs to be addressed further as part of diagnostic practice ^19, 20^.

***c) Cognitive impairments.*** Acute neurological symptoms due to NCSE also may have the appearance of acute dementia. The following cases of NCSE have been reported: normalization on the revised Hasegawa dementia scale from a score of 16 on hospitalization (due to NCSE) to 30 (full score) after antiepileptic medication in a 78-year-old woman ^21^, remission of fluctuating behavioral disturbance in response to antiepileptic medication ^22^, and disappearance of cognitive disturbance after antiepileptic medication ^23^. We believe that these cases illustrate the necessity of including NCSE as a differential diagnosis in so-called treatable dementias.

Furthermore, a review of 10 cases of sporadic Creutzfeldt–Jakob disease (CJD) suggested that CJD was not a cause of NCSE but rather NCSE was a differential diagnosis ^24^. However, we need to be aware that NCSE can also coexist with acute and chronic neurological diseases, as has been shown in the cases of acute encephalopathy and acute stroke as a manifestation of these neurological conditions ( Table 1).

### 5) Cardiac arrest

Sudden unexpected death in epilepsy (SUDEP) is a frequent cause of non-accidental, non-suicidal sudden death in patients with epilepsy. SUDEP most often affects patients with refractory epilepsy, and the cumulative risk is 12% over 40 years for those with uncontrolled childhood-onset epilepsy ^25^. The pathology of SUDEP is not yet fully understood and is thought to be multifactorial. However, “arrhythmia” and either “hypoventilation” or “hypoxia” are thought to be involved ^26^. Recently, there have been case reports of continuous ECG monitoring detecting cardiac arrest that complicated an episode of temporal lobe epilepsy, and this finding is considered a novel clinical feature of NCSE ^27, 28^. Therefore, NCSE may be involved not only in prolonged post-hyperventilation apnea but also in SUDEP.

We should also note the Mortality in Epilepsy Monitoring Unit (EMU) Study (MORTEMUS) ^29, 30^. Between 1 January 2008 and 29 December 2009, the authors of this study made a systematic retrospective survey of EMUs located in Europe, Israel, Australia, and New Zealand to retrieve data for all CRAs. EMUs from other regions were invited to report similar cases. There were 29 CRAs reported, including 16 SUDEP (14 at night), nine near SUDEP, and four deaths from other causes. Cardio-respiratory data, available for 10 cases of SUDEP, showed a consistent and previously unrecognized pattern, whereby rapid breathing (18–50 breaths/minute) developed after secondary generalized tonic-clonic seizure, followed within three minutes by transient or terminal cardio-respiratory dysfunction. Where transient, this dysfunction later recurred with terminal apnea occurring within 11 minutes of the end of the seizure, followed by cardiac arrest. SUDEP incidence in adult EMU was 5.1 per 1,000 patient-years. This study first revealed that SUDEP in EMU primarily follows an early postictal, centrally mediated, severe alteration of respiratory and cardiac function induced by generalized tonic-clonic seizure, leading to immediate death or a short period of partly restored cardio-respiratory function followed by terminal apnea and then cardiac arrest. Although small in subject number and lacking pathological data in half the cases of SUDEP as well as data on blood pressure, cerebral perfusion, oximetry, and partial pressure of CO _2_, this paper is critical and a landmark study in the management and prevention not only of SUDEP but also of sudden death in general and various acute critical conditions of unknown etiology.

So cardiac arrest might possibly be a manifestation of NCSE, although it might correspond to a postictal state electrophysiologically. Conversely, physicians should include NCSE in the causative differential diagnosis of cardiac arrest, especially of unknown cause ^31, 32^. Regarding the mechanism of SUDEP, it might be plausible that it involves derangements of the central autonomic network (CAN), which includes the insular cortex, amygdala, hypothalamus, periaqueductal gray matter, parabrachial complex, nucleus of the tractus solitarius, and ventrolateral medulla.

### 6) Autonomic dysfunction

Autonomic function is often impaired during epileptic seizures, but many such cases are mild gastrointestinal or cardiovascular autonomic events. Thus, epilepsy or NCSE could be more likely to be overlooked if autonomic impairment is regarded as primary. In Panayiotopoulos syndrome, a common idiopathic childhood-specific seizure disorder, convulsive SE is extremely rare, and autonomic symptoms may be the only features of the seizures. Half of the seizures in this syndrome last for >30 minutes, thus constituting autonomic SE ^33^.

### 7) Abnormal eye position and movement

Complex partial seizures can manifest as spontaneous nystagmus and conjugate eye deviation in addition to classical clinical features such as staring and repetitive blinking. Such manifestations can often be observed not only in patients with idiopathic epilepsy but also in critically ill patients and might suggest coexistent NCSE; however, less attention is usually paid in the latter setting. In clinical practice, we need to be aware that such findings can also be manifestations of NCSE.

### 8) Myoclonus of the face and extremities

Small amplitude myoclonus of the face and extremities is thought to be a frequently observed manifestation of NCSE. Such manifestations can also often be observed in critically ill patients and suggest coexistent NCSE; however, less attention is usually paid to this too. In clinical practice, we need to be aware that such findings can also be manifestations of NCSE.

### 9) Miscellaneous signs and symptoms

We have treated patients with mimetic facial automatism or recurrent attacks of unconsciousness, both of which were manifestations of NCSE and both of which disappeared immediately in response to antiepileptic medication ( Table 1). In the literature, NCSE can manifest as persistent laughing (status gelasticus), vertigo, or dizziness ^34, 35^, which might be consistent with transient neurological attack (TNA) ^32^. In general, neurological deficits of an unexplained, episodic, fluctuating, or recurrent nature should arouse suspicion of NCSE.

### 10) Acute organ dysfunction

To elucidate the relationship between SE and acute organ dysfunctions (ODs), we retrospectively investigated 30 patients with SE (from April 2006 to March 2013, 2.9% of all inpatients) for clinical features including first-ever ODs which were complicated just after ictus. Generalized convulsive SE (GCSE) was seen in five patients (mean 64.6 years old), NCSE was seen in 15 patients (mean 70.5 years old; complex partial SE in 14 and absence SE in one), and both GCSE and NCSE during the attack temporally apart were seen in 10 patients (mean 54.1 years old). ODs were observed in three GCSE patients (60%, multiple organ failure, arrhythmia, and liver dysfunction), six NCSE patients (40.0%, acute respiratory failure, alveolar hypoventilation, acute cardiopulmonary arrest, acute takotsubo cardiomyopathy, renal dysfunction, and QT interval prolongation), and six patients with both (60%, renal dysfunction, multiple organ failure, and disseminated intravascular coagulation with neurogenic diabetes insipidus). Underlying diseases in those patients with OD were acute encephalopathy in two, acute encephalomyelitis in two, cerebral infarction in two, acute cerebral sinus occlusion in one, and senile dementia of the Lewy body type in one; there was no underlying disease in one patient. Mortality at discharge was 33% and 9.1% in those patients with or without ODs, respectively.

One must be careful about interpreting acute ODs because some might reflect postictal secondary complications unrelated to the epileptic attack itself. However, we defined acute ODs as those first-ever ODs which were complicated just after the ictus, which may eliminate the likelihood of secondary complication to a good degree. So we proposed the novel concept of epilepsy-related organ dysfunction (Epi-ROD), i.e. critical complications of convulsive and nonconvulsive SE ^29, 30^. Features of Epi-ROD can be summarized as follows: 1) frequently observed in both convulsive SE and NCSE (convulsive SE 60%, NCSE 40%, and both 60%), 2) life-threatening with high mortality (33.3%), 3) can be observed in those with acute encephalopathy, stroke, CNS infection, and so on, and 4) heterogeneous in nature. The causal relationship between Epi-ROD and epileptic attack needs to be explored in larger subjects. Vice versa, most importantly, we need to consider convulsive SE and especially NCSE in the differential diagnosis of various acute ODs, even in the absence of overt seizures ( Table 3). Also, we always need to be cautious about the relationship between cause and effect.

**Table 3.** Epilepsy-related organ dysfunction (Epi-ROD).

Features	Frequent in both convulsive status epilepticus (SE) and nonconvulsive status epilepticus (NCSE) Convulsive SE 60%, NCSE 40%, both 60% Life-threatening/high mortality (33.3%) with acute encephalopathy, stroke, and central nervous system infection, and so on Heterogeneous in nature
Implication	Differentiate SE in acute OD, even without overt seizure

OD: organ dysfunction

## Ongoing issues

Following the new definition of SE by the Neurocritical Care Society in 2012, patients with SE are increasingly recognized globally ^3^. Considering the fact that NCSE is more frequent than GCSE, SE is the most frequent neurologic complication of critical medical illnesses. However, the clinical features of NCSE are not yet well recognized by most clinicians. Therefore, it is important to include NCSE in undergraduate and postgraduate medical education in related disciplines.

NCSE is a potentially treatable condition, although treatment strategies and guidelines are not firmly established yet. The underdiagnosis of NCSE is due to 1) lack of knowledge of NCSE itself, 2) lack of recognition about the diversity of NCSE, and hence attribution of the impaired state to other causes (e.g. metabolic encephalopathy or postictal state), and 3) lack of an appropriate screening tool (EEG) for NCSE available anytime, anywhere, under any conditions, and to anyone. Recently, we created a novel electrode and headset which enables prompt and continuous EEG monitoring from the ER to the neuro-ICU ^36^. Prompt EEG monitoring would improve the diagnosis of NCSE and might further expand the clinical spectrum of NCSE. Along with this device, there is an urgent need for formal, global, and practical criteria for NCSE.
